# PHGDH-mediated serine synthesis reduces oligodendrocyte death by sustaining GSH and NADPH levels after brain ischemia

**DOI:** 10.3389/fphar.2026.1735477

**Published:** 2026-04-15

**Authors:** Zhaolong Zhang, Jinhua Tan, Mengfei Lv, Zhongying Duan, Congxiao Wang, Mingchen Gao, Yu Cui, Yugong Feng

**Affiliations:** 1 Department of Interventional Radiology, The Affiliated Hospital of Qingdao University, Qingdao, China; 2 Qingdao Medical College, Qingdao University, Qingdao, China; 3 Department of Neurosurgery, The Affiliated Hospital of Qingdao University, Qingdao, China

**Keywords:** ischemic stroke, OGD/R, oligodendrocytes, PHGDH, serine, white matter

## Abstract

**Introduction:**

White matter is vulnerable to early ischemic injury. The susceptibility of oligodendrocytes to ischemic damage can lead to demyelination, axonal degeneration, and neurological deficits, making them a key focus for understanding and developing therapies for stroke-related brain injury. In this study, we aimed to investigate how cell metabolism contributes to oligodendrocyte survival and white matter integrity maintenance following ischemia.

**Methods:**

NCT-503 was injected after inducing transient middle cerebral artery occlusion. After reperfusion, brain infarct volume and neurobehavioral deficits and behaviour performance were assessed. Immunofluorescence staining was performed to evaluate oligodendrocytes death. Cell viability was measured using the CCK8 assay. Flow cytometry analysis was conducted to examine reactive oxygen species (ROS) levels.

**Results:**

*De novo* serine synthesis pathway enzyme phosphoglycerate dehydrogenase (PHGDH) was hardly expressed in neurons, microglia and oligodendrocyte precursor cells (OPCs) but selectively expressed in mature oligodendrocytes and astrocytes. Brain ischemic injury specifically enhanced the expression of PHGDH in oligodendrocytes. PHGDH inhibition with NCT-503 did not affect acute neuronal injury but worsened sensorimotor and cognitive functional outcomes after ischemic stroke. Moreover, white matter integrity and oligodendrocyte survival were specifically reduced after PHGDH inhibition and serine supplementation facilitated oligodendrocyte survival and enhanced white matter integrity, and consequently improved neurological functions. Mechanistically, PHGDH-mediated serine synthesis protected oligodendrocytes from oxidative stress-induced death by promoting glutathione (GSH) and nicotinamide adenine dinucleotide phosphate (NADPH) production through the one-carbon metabolism pathway.

**Conclusion:**

This study reveals the role of PHGDH-mediated *de novo* serine synthesis in reducing oligodendrocyte death which may provide a potential target for improving neurological function after ischemic stroke.

## Graphical Abstract

**Figure:**
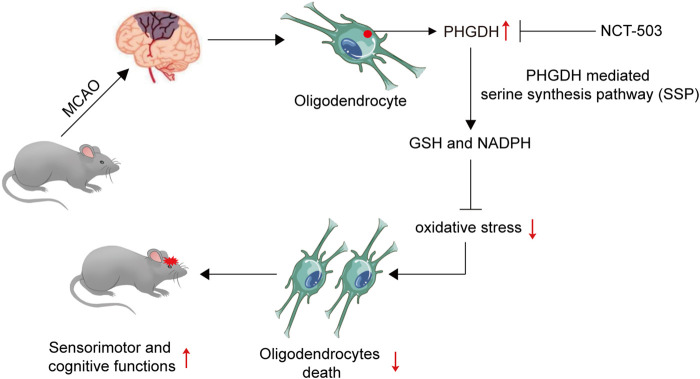

## 1 Introduction

Stroke is a leading cause of permanent disability and mortality worldwide. Ischemic stroke accounts for more than 80% of all strokes (Feske, 2021; Tu and Wang, 2023). Both white matter and grey matter are vulnerable to early ischemic injury (Ho et al., 2005; Stebbins et al., 2008). White matter is mainly composed of axons, myelin-producing oligodendrocytes (OLs), and other glial cells (Liu et al., 2017). Injured white matter exhibits oligodendrocyte abnormalities and loss of the myelin sheath can be observed within days after ischemic stroke (Pantoni et al., 1996; Wang et al., 2016). The resulting disruption of white matter integrity leads to long-term functional and cognitive deficits after ischemic stroke (McIver et al., 2010; Wang et al., 2016). Although ischemic brain engages repair mechanisms by the proliferation and differentiation of oligodendrocyte precursor cells (OPCs) into mature oligodendrocytes to restore white matter integrity (Zhang et al., 2013), the maintenance of mature oligodendrocytes viability after ischemic injury is also vital for the repair of brain white matter (Dai et al., 2020; Dewar et al., 2003). Uncovering the molecular mechanism of how oligodendrocytes preserve their viability after ischemic stroke is important for the design of drugs to improve neurological function. To date, several factors have been identified as contributing to OL survival, such as high-mobility group box-1 (HMGB1) (Choi et al., 2017), transforming growth factor a (TGFα) (Dai et al., 2020) and interleukin 33 (IL-33) (Xie et al., 2021). However, the role of cellular metabolism in regulating oligodendrocytes survival after ischemic stroke remains poorly understood.

Serine can either be taken up from the extracellular microenvironment or synthesized *de novo* in the cytoplasm (Shen et al., 2021). Exogenous serine can be transported into cells via membrane transport systems, such as ASCT1/2 (Slc1a4) and ASC-1 (Jiang et al., 2020). Under physiological conditions, the *de novo* serine synthesis pathway represents the main source of serine. Phosphoglycerate dehydrogenase (PHGDH) catalyzes the first step of this pathway by catalyzing the oxidation of 3-phosphoglycerate (3-PG) derived from glycolysis to 3-phosphohydroxypyruvate (3-PHP) by NAD^+^-coupled redox reactions (Klomp et al., 2000; Reid et al., 2018). PHGDH-mediated synthesis of serine contributes to tissue development (Stegen et al., 2022; Vandekeere et al., 2018; Wang et al., 2022) and disease progression, such as metabolic diseases (Chen H. et al., 2022; Sim et al., 2020), immune-related diseases (Shan et al., 2022; Shen et al., 2021) and neurodegeneration (Chen et al., 2025; Lv et al., 2025). Previous studies have shown that supplemental L-serine exerts neuroprotective effects through multiple mechanisms, such as promoting neuronal synaptic activity (Wang et al., 2010; Ye et al., 2021), enhancing cerebral blood flow (CBF) (Ren et al., 2013), facilitating OPCs proliferation and the survival and differentiation of neural stem cells (NSCs) (Metcalf et al., 2018; Sun et al., 2014). In addition, PHGDH-mediated serine synthesis has been reported to regulate astrocyte-medaited neuroinflammation (Chen et al., 2025; Lv et al., 2025). However, the role of PHGDH-mediated serine *de novo* synthesis in ischemic-induced neuronal or oligodendrocytes injury remains unknown.

In this study, we found that cerebral ischemic injury increased the expression of PHGDH in oligodendrocytes. Further investigation indicated that PHGDH-mediated generation of serine directly preserved oligodendrocytes survival by facilitating GSH and NADPH production via one carbon metabolism, thereby counteracting oxidative stress after ischemia and ultimately improving neurological function.

## 2 Materials and methods

### 2.1 Mice and focal cerebral ischemia

9–10 weeks C57BL/6 male mice were used for transient middle cerebral artery occlusion (tMCAO). Male mice were purchased from Jinan Pengyue Experimental Animal Breeding Company (China). All animal experiments comply with National Institutes of Health guidelines and were approved by the institutional animal care and use committee of Qingdao University (QDU-AEC-2024002).

Transient focal cerebral ischemia was conduced through suture occlusion as previously reported (Cui et al., 2024). 9–10 weeks C57BL/6 mice (male) were anesthetized with 4% isoflurane in 70% N_2_O and 30% O_2_. The left middle cerebral artery was obstructed by inserting a monofilament nylon suture from the left external carotid artery (ECA) into the left internal carotid artery. After occlusion for 1.5 h, the suture was removed to recover the blood flow. Laser Doppler flowmetry (PERIMED PSI-Z) was used to monitor the blood flow of the mice brains. For the sham-operated group, the mice underwent the same surgery except for insertion and removal of sutures. During the whole procedure, a warming pad (RWD, 69003) was used to maintain the temperature and a breathing machine was used to monitor the respiration of mice. Mice were placed to heated cages during the recovery phase with free access to chow food and water. After ischemia, mice without significant decrease in cerebral blood flow were excluded. After about 24 h aftr reperfusion, we evaluated neurological deficits scores and mice that showed no signs of neurological deficits were excluded from further study. To accurately assess the results, mice were randomized into different treatment groups by one investigator. The other investigator was responsible for the surgical procedure and analyzed data (Cui et al., 2024). The experiments above were performed according to the recommendations for research in experimental stroke studies and the current ARRIVE (Animal Research: Reporting of *in vivo* Experiments). Mice were excluded from the study if they died during the surgical procedure, or were euthanized due to an insufficient neurological deficit (modified Neurological Severity Score ≤2, indicating inadequate ischemia), or because the regional cerebral blood flow (rCBF) during MCAO decreased by less than 65% (Zhang et al., 2018). A total of 182 mice underwent MCAO surgery; Of these, 55 mice were excluded for the following reasons: death during surgery, mNSS ≤2, rCBF decrease <65% during occlusion.

### 2.2 *In vivo* drug delivery

After tMCAO treatment, 9–10 weeks C57BL/6 mice were injected with drugs, and the model mice were randomly grouped before injection. Intraperitoneal injections of the corresponding drugs were administered at 6 h, 24 h, 48 h and 72 h after tMCAO for behavior analysis. For brain infarction analysis, intraperitoneal injections of the corresponding drugs were administered at 0 h and 6 h after reperfusion. The experimental group received an intraperitoneal injection of NCT-503 at a concentration of 20 mg/kg, the control group received the same dose of solvent (5% ethonal +35% PEG300 + 60% of aqueous 30% 2-Hydroxypropyl-β-cyclodextrin), and the serine was injected at a concentration of 100 mg/kg in saline.

### 2.3 TTC staining and neurological severity score analysis

Mice were euthanized at 24 h by isoflurane overdose and the brain were harvested and TTC (2,3,5-triphenyltetrazolium chloride) staining was performed. The brain was placed in a 6 cm Petri dish and frozen in minus 80° and then cut into 1.2 mm coronal slices and incubated for 30 min at 37 °C with 2% TTC in phosphate buffered saline (PBS). Image acquisition, processing and analysis were performed blindly. The scanned images were analyzed by using image analysis software (ImageJ). To calculate infarct volume, edema was first corrected. The normal volume of the contralateral hemisphere and the ipsilateral hemisphere were measured and the percent infarction was calculated as % contralateral structure to avoid false positive secondary to edema (Cui et al., 2021; Cui et al., 2024).

Neurological deficits were tested using the a nine points scoring systems: 0 point, normal behavior; one point, left forelimb flexion upon suspension by the tail or failure to fully extend the right forepaw; two points, left shoulder adduction upon suspension by the tail; three points, reduced resistance to a lateral push toward the left; four points, circling to the left only if pulled by the tail and spontaneous movement in all directions; five points, walking or circling spontaneously only to the left; six points, walking only after stimulation; seven points, no spontaneous behavior to stimulation; and eight point, stroke-induced death (Cui et al., 2021).

### 2.4 Mice behavior analysis

The adhesive removal test, rotarod test and novel object recognition test were performed prior to tMCAO (one or 2 days before surgery) and on days 4, 7, and 14 after tMCAO. The adhesive removal test was performed to assess sensorimotor asymmetries. Two 2 × 3 mm adhesive tapes were applied to lesioned forepaws and healthy forepaws. Tactile responses were measured by recording the time to remove the adhesive tape, with a maximum observation period of 120 s.

For the rotarod test, mice were trained twice daily (10 min per session) for three consecutive days at the speed of 10 rpm before tMCAO. For the formal test, mice were placed on a rotating drum with speeds starting at 4 rpm and accelerating to 45 rpm within 240s. Three times daily to assess overall coordination and balance. The Rota-Rod test apparatus was used (HUAYO-NHR420).

The novel object recognition test. On the first day, the mice were habituated for 10 min in an empty open arena. On the second day, two similar objects were positioned in different corners of the arena at equal distances from the walls, and mice were placed inside and given a duration of 10 min for exploration. After that, mice were placed back into their home feeding cage for a 1-h interval. Furthermore, one of the objects was then substituted with a new object, and the mice were again placed in the arena and allowed to explore for 10min. The total time spent exploring each object (novel and familiar) was recorded. The discrimination index was calculated as the difference between the percentages of time spent investigating the novel object and the time spent investigating the familiar object plus novel object. All trials were video-recorded (TopScan, Clever Sys) and scored by researchers who remained blinded to experimental conditions in order to ensure accuracy (Bu et al., 2024; Chindo et al., 2024; Pan et al., 2024).

### 2.5 Antibodies and reagents

The following antibodies were used: PHGDH antibody (14719-1-AP, Proteintech), SLC1A4 antibody (13067-2-AP, Proteintech), β-actin (66009-1-Ig, Proteintech), Phospho-SAPK/JNK (Thr183/Tyr185) (81E11) Rabbit mAb (4668), SAPK/JNK Antibody (9252), Phospho-p44/42 MAPK (Erk1/2) (Thr202/Tyr204) Antibody (9101S), and p44/42 MAPK (Erk1/2) Antibody (4695), HRP-conjugated Affinipure Goat Anti-Mouse IgG (H + L) (SA00001-1, Proteintech), HRP-conjugated Affinipure Goat Anti-Rabbit IgG (H + L) (SA00001-2, Proteintech). NCT-503 (T4213, Topscience, 20 μM), SAM (S5109, Selleck, 400 μM), Na-Formate (247596, sigma, 1 mM), NAC (T0875, Topscience, 10 mM), serine (T2O2730, Topscience), Phorbol 12-myristate 13-acetate (Sigma-Aldrich, P1585), NADPH (ST360, Beyotime).

### 2.6 Luxol fast blue myelin (LFB) staining

The demyelinating lesions in mouse brain tissue were detected using Luxol Fast Blue Myelin Stain Kit (Eosin Method) (G3240, Solarbio). Briefly, after washing with PBS, frozen brain slices were incubated overnight in solid blue staining solution at room temperature, and then washed in 95% ethanol to remove excess staining solution. After rinsing with distilled water, the color was separated in solid blue differentiation solution for 15s. After that, the color was separated in 70% ethanol for 30 s until the gray matter was clear. After rinsing with distilled water, it was re-dyed in eosin dyeing solution for 30 s–1 min, and then washed quickly. After differentiation, the brain sections were quickly dehydrated with 95% and 100% ethanol, transparent by xylene, and then sealed with neutral gum. Slides were taken using a Nikon upright fluorescence microscope (Ni-U).

### 2.7 Cell culture and OGD treatment

Cortical neurons were isolated from the cortex of C57BL/6 mouse embryos at embryonic day 17 (E17), as previously described (Cui et al., 2021). The meninges were removed and the dissected cortices were subjected to trypsin digestion (0.05%) and trituration to obtain a single-cell suspension. Subsequently, the cells were plated at a density of 0.2 ×10^6^ in poly-D-lysine-coated plastic 24-well plates using neurobasal feeding medium (Neurobasal medium-GIBOCO, 2% B-27 supplement-GIBOCO, 100 U/mL penicillin, and 100 μg/mL streptomycin, 0.5 mM L-glutamine and 25 mM glutamic acid). The cells were cultured at a temperature of 37 °C under a humidified atmosphere containing 5% CO2. Culture media were refreshed every 3 days. After incubation for a period of 9–11 days, the cultured neurons were utilized for subsequent experiments.

MO3.13 cell line was purchased from Procell (CL-0072, Wuhan, China). The cells were cultured with DMEM medium containing 10% FBS. All cells were maintained at 37 °C in a humidified incubator containing 5% CO2. Cells were differentiated with 100 nM Phorbol 12-myristate 13-acetate (PMA, Sigma-Aldrich, P1585) for 2 days (Boscia et al., 2012).

For the OGD challenge, neurons or MO3.13 cells were transferred to deoxygenated glucose-free extracellular solutions; placed into the anaerobic incubator (RUSKIN, United Kingdom) and held in 95% N2/5% CO2 at 37 °C for 4.5 h. The medium was then replaced with fresh normal culture medium for the indicated times during recovery in a 95% O2/5% CO2 incubator at 37 °C (Cui et al., 2024).

### 2.8 Immunofluorescence

Experimental animals were anesthetized and perfused with 0.9% saline followed by 4% paraformaldehyde. Brains are harvested and fixed in 4% PFA in phosphate buffer for 12 h at room temperature (RT) and incubated at 4 °C in phosphate buffer containing 30% sucrose for 48 h. The brains were frozen in optimum cutting temperature compound and sectioned at 40 μm, then processed for immunofluorescence staining. The brain slices were washed with PBS, perforated with 0.3% Triton X-100 for 30 min at room temperature and blocked with 10% indicated serums for 2 h at room temperature. Sections were then incubated with primary antibodies and secondary antibodies. The nuclei were stained with DAPI (S2110, Solarbio). The following antibodies were used: rabbit anti-PHGDH (14719-1-AP, Proteintech, 1:1000), mouse anti-NeuN (MAB377, Millipore, 1:500), mouse anti-GFAP (MAB360, Millipore, 1:1000), goat anti-Iba-1 (NB100-1028, Novus, 1:1000), PDGFRα (AF1062, R&D, 1:1000), APC (GTX16794, Gentex, 1:100), MBP (ab40390, abcam, 1:1000), SMI32 (801701, Biologend, 1:500). Donkey anti-rabbit 488 (A21206), Donkey anti-mouse 568 (A10037) and Donkey anti-goat 568 (A11057) from Thermo Fisher Scientific. All the slides were taken with a confocal microscope Nikon C2 (Nikon-Ti2-E) and analyzed using ImageJ software.

### 2.9 Measurement of intracellular ROS

The intracellular ROS levels were detected using a peroxide-sensitive fluorescent probe (DCFH-DA; Beyotime, China) according to the instructions of the manufacturer. Briefly, DCFH-DA was diluted at a final concentration of 10 μM using fresh DMEM/F12. Remove the cell culture medium, add an appropriate volume of diluted DCFH-DA and incubate with cells for 30 min at 37 °C. The cells were then harvested and washed twice with PBS. Cell fluorescence was acquired on a BECKMAN CytoFLEX S flow cytometer and analyzed with FlowJo software.

### 2.10 siRNA-mediated cell transfection

To silence the expression of PHGDH, 20 nM siNC and siPHGDH siRNA were transfected into MO3.13 cells using standard procedures with Lipofectamine RNAiMAX Transfection Reagent. Cells were harvested for RNA expression analysis at 48 h after transfection. The following siRNA sequences were used: mouse PHGDH sense: 5′-UCG​GCA​GAA​UUG​GAA​GAG​Att-3’; mouse PHGDH anti-sense: 5′- UCU​CUU​CCA​AUU​CUG​CCG​Att-3’.

### 2.11 Statistical analysis

The sample size required for the animal study was empirically determined based on the results of previous experiments and similar to the size generally used in the field. All data were shown as Mean ± S.D., which was chosen to describe the variability of the observed data in each group, consistent with field standards. The statistical analysis was performed using GraphPad Prism software. All experiments were performed three or more times unless otherwise indicated. To compare the statistical significance of two independent groups, Student’s t-test (two-tailed) was used unless otherwise indicated. Differences between multiple groups were analyzed by one-way or two-way ANOVA followed by indicated test shown in the Figure legends.

## 3 Results

### 3.1 PHGDH shows oligodendrocyte-specific upregulation after brain ischemic injury

**FIGURE 1:**
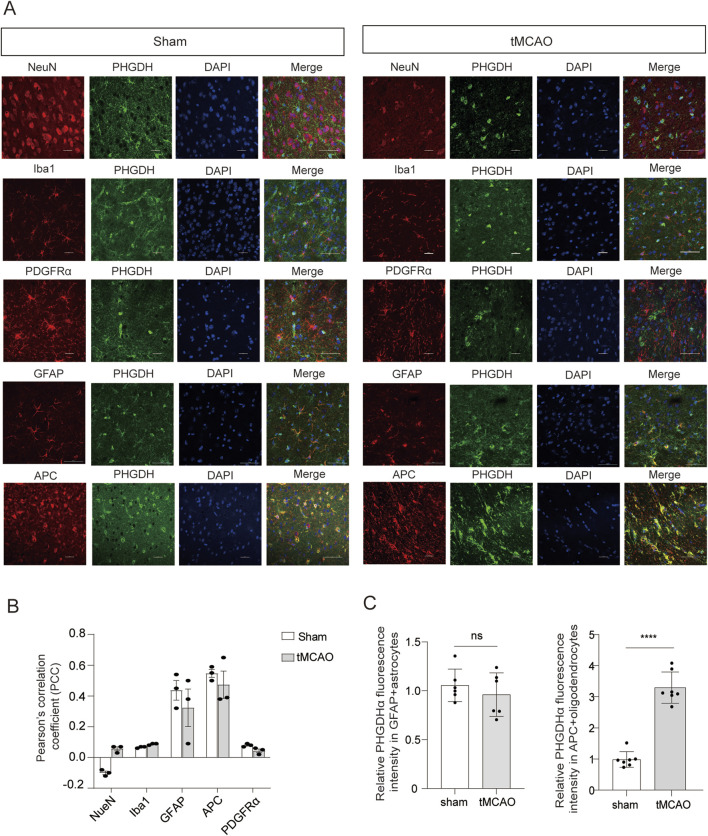
The expression of PHGDH was selectively upregulated in oligodendrocytes after ischemic brain injury. **(A)** Representative confocal images of PHGDH expression in brain cells of C57BL/6 mice. Scale bar, 10 μm. n = 4 mice per group. **(B)** Quantification of co-localization of NeuN, GFAP, Iba1, PDGFRα and APC with PHGDH in tMCAO (ischemia) or sham-operated brains, calculated as Pearson’s correlation coefficient, r. n = 3 mice per group. **(C)** Quantification of PHGDH abundance in GFAP^+^ astrocytes (left, n = 5 mice per group) and APC^+^ oligodendrocytes (right, n = 7 mice per group) in tMCAO (ischemia) or sham-operated brains. The data are means ± S.D., for all panels: *P < 0.05, **P < 0.01, ***P < 0.001, n. s., no significance by Two-Way ANOVA analysis followed by Bonferroni Test **(B)** and Student’s t-test **(C)**. All data are representative of or combined from at least three independent experiments.

To investigate the role of PHGDH-mediated *de novo* serine synthesis in ischemia-induced brain injury, we examined the relative abundance of PHGDH in different cell types of C57BL/6 mice brains either after ischemic injury or sham-operation by using immunofluorescent staining. Interestingly, PHGDH was barely colocalized with cortical neurons (NeuN^+^), microglia (Iba-1^+^) and OPCs (PDGFRα^+^) in brains collected 24 h after ischemia or in sham-operated controls. In contrast, a substantial colocalization of PHGDH with APC^+^ oligodendrocytes and GFAP^+^ astrocytes was observed in both sham and ischemic brains (Figures 1A,B), indicating that PHGDH was selectively expressed in oligodendrocytes and astrocytes.

To further determine whether ischemic injury increased PHGDH expression in oligodendrocytes and astrocytes, we quantified PHGDH fluorescence intensity in APC^+^ and GFAP^+^ cells from sham and tMCAO groups. Notably, cerebral I/R injury significantly increased PHGDH expression in APC^+^ oligodendrocytes compared with sham controls, whereas PHGDH levels in astrocytes was not altered (Figure 1C). These findings demonstrate that PHGDH was selectively upregulated in oligodendrocytes following cerebral ischemic injury.

### 3.2 Inhibition of PHGDH exacerbates functional outcomes after brain ischemic injury

**FIGURE 2:**
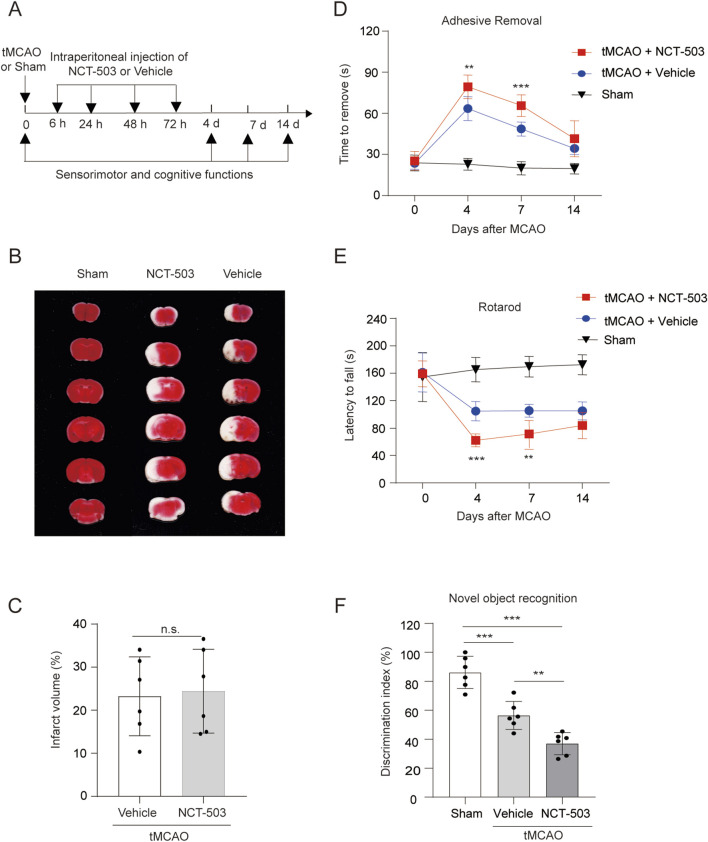
PHGDH inhibition exacerbates neurological function after brain ischemia. **(A)** Schematic of experimental timeline. **(B,C)** Representative image **(B)** and quantification **(C)** of brain slices stained by TTC at 24 h after tMCAO in vehicle or NCT-503 injected mice. n = 6 per group. **(D–F)** PHGDH inhibition deteriorates long-term neurological deficits after tMCAO as assessed by adhesive removal test **(D)**, rotarod tests **(E)** and NOR test **(F)**. n = 6 for each group. The data are means ± S.D. for all panels: ***P < 0.001, n. s., no significance by Student’s t-test **(C)** and One-way ANOVA analysis was used followed by Tukey’s test **(D–F)**. All data are representative of or combined from at least three independent experiments.

To test the effects of PHGDH on brain ischemic injury *in vivo* conditions, we used NCT-503, a well-established specific inhibitor of PHGDH (Pacold et al., 2016), to inhibit PHGDH activity, and examined long-term functional outcomes after brain ischemia. NCT-503 or vehicle treatment did not alter sensorimotor and cognitive function in physiological conditions (Supplementary Figure S1A–D). TTC staining confirmed that PHGDH inhibition with NCT-503 failed to affect infarct volume during the acute phase of ischemic stroke (Figures 2A–C), which is consistent with the lack of PHGDH expression in neurons observed in Figure 1A. To further assess the direct effects of PHGDH inhibition on neurons, we cultured cortical neurons and subjected neurons to oxygen-glucose deprivation followed by reoxygenation (OGD/R), an *in vitro* treatment that mimics the pathways associated with brain ischemia and examined neuronal viability. Consistent with the *in vivo* findings, inhibition of PHGDH with NCT-503 in primary cultured neurons did not alter neuronal viability either under normal conditions or after OGD/R treatment (Supplementary Figure S2A). Therefore, PHGDH did not appear to directly influence neuronal death after ischemic brain injury.

Considering the specific upregulation of PHGDH in oligodendrocytes and the known role of oligodendrocytes in neurological function, we next analyzed the functional recovery in vehicle and NCT-503-treated mice after cerebral ischemia. In the adhesive removal test, PHGDH inhibitor-treated mice required more time to remove adhesive strips from their paws than vehicle-treated controls at 4 and 7 days after ischemic injury (Figure 2D). Moreover, motor coordination, as assessed by the rotarod test, was impaired in PHGDH-inhibited mice, which exhibited significantly shorter latencies to fall at both 4 and 7 days post-injury (Figure 2E). Cognitive function, tested by novel object recognition, was also decreased in NCT-503-treated mice compared with vehicle controls at 4 and 7 days following ischemic injury (Figure 2F). Together, inhibition of PHGDH exacerbated sensorimotor and cognitive function outcomes following ischemic brain injury.

### 3.3 Inhibition of PHGDH displays decreased white matter integrity after ischemic brain injury

**FIGURE 3:**
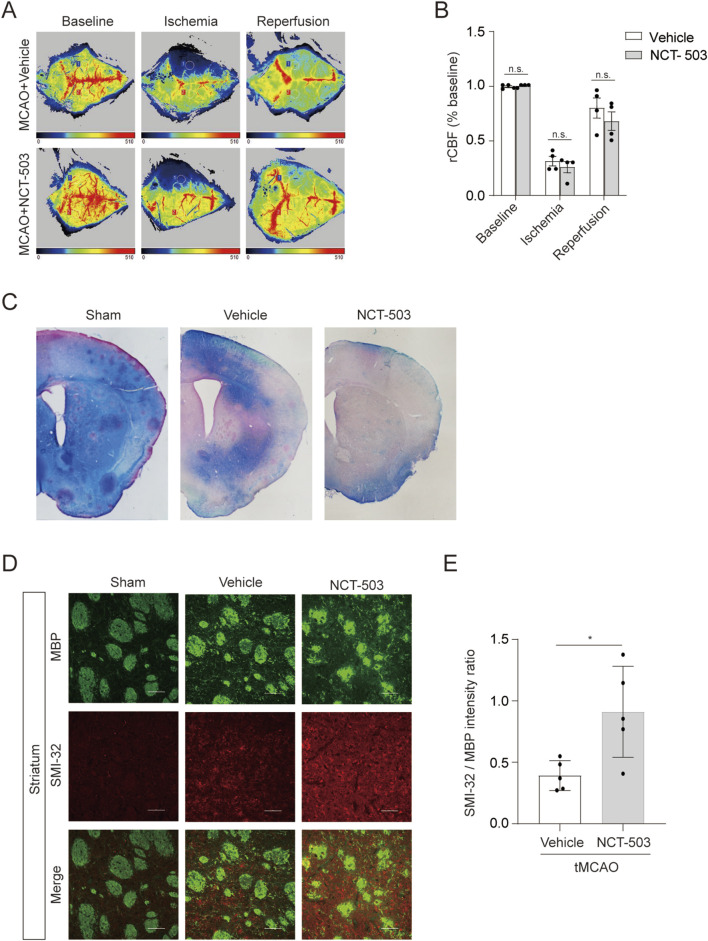
Inhibition of PHGDH displays decreased white matter integrity after ischemic brain injury. **(A,B)** Representative images **(A)** and statically analysis **(B)** of the cerebral blood flow (CBF) at different times between NCT-503-treated and control groups. n = 4 per group. **(C)** Representative images of Luxol Fast Blue staining (LFB) in the peri-infarct striatum. n = 3 per group. **(D,E)** Representative **(D)** images and quantification **(E)** of double immunostaining for myelin basic protein (MBP; green) and SMI-32 (red) in the striatum of mice subjected to tMCAO for 7 days. Scale bars = 50 μm. n = 5 for each group. The data are means ± S.D., for all panels: *P < 0.05, **P < 0.01, ***P < 0.001, n. s., no significance by Two-Way ANOVA analysis followed by Bonferroni Test **(B)** and Student’s t-test **(E)**. All data are representative of or combined from at least three independent experiments.

To determine why NCT-503 treatment exacerbated functional recovery, we first tested whether inhibition of PHGDH affected the cerebral vasculature. A significant reduction in cerebral blood flow (CBF) measured by laser Doppler flowmetry after ischemia compared with that in normal mice confirmed the successful establishment of tMCAO models (Figure 3A). No significant differences in CBF were observed between vehicle or NCT-503-treated mice either during ischemia or after reperfusion (Figures 3A,B). Thus, PHGDH inhibition did not alter cerebral blood flow following ischemic injury.

We next investigated the effects of NCT-503 on astrocytes. Administration of NCT-503 did not decrease astrocytes vialibity (Supplementary Figure S3A). In addition, the activation of astrocytes was reduced in NCT-503-treated mice (Supplementary Figure S3B), indicating that the worsened functional outcomes were unlikely to result from direct effects of NCT-503 on astrocytes.

Given that mature oligodendrocytes are vulnerable to ischemic injury and the resultant demyelination decreases white matter integrity, we wondered whether the reduced long-term sensory motor function and cognitive function in ischemic mice resulted from compromised white matter integrity. PHGDH inhibition with NCT-503 increased myelin loss 7 days after ischemic injury, as determined by luxol fast blue (LFB) staining (Figure 3C). Consistently, double immunofluorescent staining showed that white matter integrity within the striatum—assessed as the ratio of SMI32 to MBP immunofluorescence intensity—was significantly reduced at day 7 after ischemic stroke in NCT-503–treated mice compared with vehicle controls (Figures 3D,E). These findings indicate that PHGDH inhibition impaired white matter integrity after ischemic brain injury.

### 3.4 PHGDH inhibition aggravates oligodendrocytes death after ischemic brain injury

**FIGURE 4:**
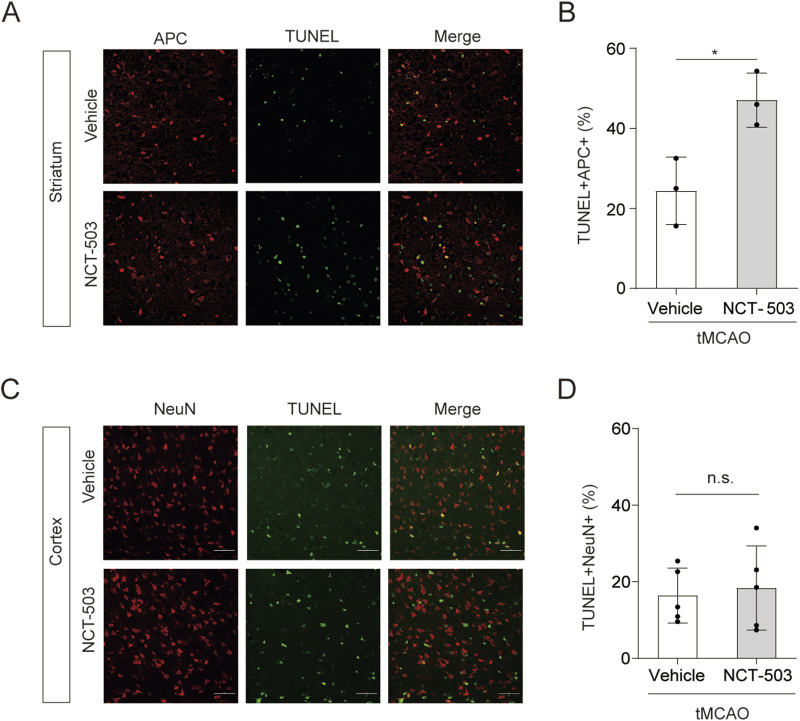
Inhibition of PHGDH decreases oligodendrocyte survival after ischemic brain injury. **(A,B)** Representative images **(A)** and statistical analysis **(B)** of tunnel^+^ APC^+^ cells in the striatum of ischemia brains at 3 days after tMCAO between NCT-503-treated and control groups. n = 3 per group. **(C,D)** Representative images **(C)** and statistical analysis **(D)** of tunnel^+^ NeuN^+^ cells in the cortex of ischemia brains at 3 days after tMCAO between NCT-503-treated and control groups. n = 5 per group. The data are means ± S.D. for all panels: ***P < 0.001, n. s., no significance by Student’s t-test **(B,D)**.

As PHGDH was more prominently expressed in oligodendrocytes (and astrocytes) compared with neurons, microglia, and OPCs, we first measured whether inhibition of PHGDH affected death of oligodendrocytes. At day 3 after ischemic brain injury, PHGDH inhibition significantly increased oligodendrocyte death in the striatum, as indicated by TUNEL^+^/APC^+^ staining, compared with vehicle-treated controls (Figures 4A,B). To further exclude the possibility that impaired white matter integrity was caused by increased neuronal death, we also assed neuronal death. As predicted, the death of cortical neurons (marked by TUNEL^+^/NeuN^+^ staining) was not significantly influenced between NCT-503-treated and vehicle-treated mice (Figures 4C,D). Thus, inhibition of PHGDH aggravated oligodendrocyte death during the acute phase after ischemic stroke.

### 3.5 Serine supplementation rescues the increased demyelination after ischemic brain injury

**FIGURE 5:**
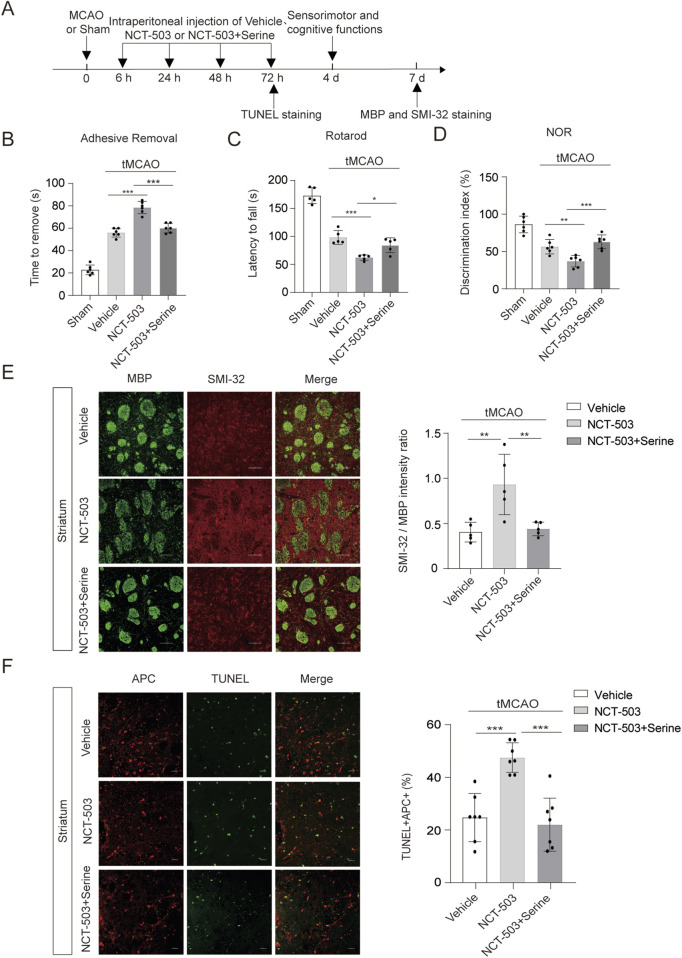
Supplementation of serine rescues the increased demyelination after brain ischemia. **(A)** Schematic of experimental timeline. **(B–D)** Serine administration in the presence of NTC-503 alleviates long-term neurological deficits after tMCAO as assessed by adhesive removal test, rotarod test and NOR test. n = 6 **(B,D)** and n = 5 **(C)** for each group. **(E)** Representative images and quantification of double immunostaining for myelin basic protein (MBP; green) and SMI-32 (red) in the striatum of mice subjected to tMCAO for 7 days. Scale bars = 50 μm. n = 5 for each group. The vehicle group and NCT-503 group data shown in Figure 3D and **(E)** are statistically derived from the same set of animals. **(F)** Representative images and statistical analysis of tunnel^+^ APC^+^ cells in the cortex of ischemia brains at 3 days after tMCAO in different treatment groups. n = 7 per group. The data are means ± S.D. for all panels: ***P < 0.001, n. s., no significance by One-way ANOVA analysis was used followed by Tukey’s test **(B–F)**.

As PHGDH is vital for serine synthesis, we investigated whether supplementation with serine could reverse the increased demyelination caused by PHGDH inhibition during ischemic brain injury. As predicted, intraperitoneal injection of NCT-503 excerbated both sensorimotor and cognitive functional deficits after ischemic brain injury, whereas co-administration of NCT-503 and L-serine significantly improved these outcomes. Specially, L-serine treatment in the presence of NCT-503 reduced the time to remove stickers (Figures 5A,B), increased the latency to fall in the roartord test (Figure 5C) and enhanced performance in the novel objection recognition test (Figure 5D). These results indicate that L-serine supplementation reversed the neurological functional deficits induced by PHGDH inhibition.

We next examined whether serine supplementation could also rescue white matter integrity and reduce oligodendrocytes death. Inhibition of PHGDH reduced white matter integrity as reflected by an increased SMI32/MBP immunofluorescence intensity ratio at day 7 (Figure 5E) and cotreatment with L-serine improved white matter integrity after ischemic brain injury (Figure 5E). Moreover, the increased frequency of apoptotic oligodendrocyets observed in NCT-503-treated mice was reversed by L-serine supplementation (Figure 5F). Collectively, these data demonstrate that PHGDH-mediated serine synthesis protects against oligodendrocyte death following ischemic brain injury.

### 3.6 PHGDH-mediated serine synthesis directly reduces oligodendrocytes death *in vitro*

**FIGURE 6:**
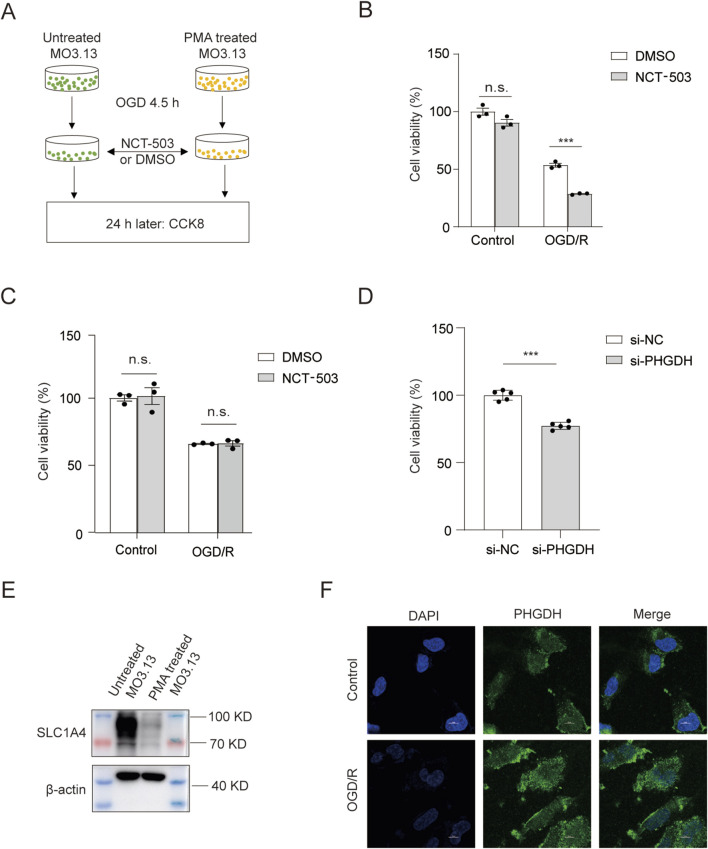
PHGDH-mediated serine synthesis directly facilitates oligodendrocyte survival *in vitro*. **(A)** Schematic of experimental timeline. **(B,C)** Cell viability of MO3.13 cells induced by PMA **(B)** or undifferentiated **(C)** in normal conditions or after OGD/R treatment in the presence of DMSO or NCT-503 treatment. n = 3 for each group. **(D)** Cell viability of MO3.13 cells induced by PMA after OGD/R treatment after transfection with siNC or siPHGDH for 2 days. n = 5 for each group. **(E)** Representative immunoblot of SLC1A4 in undifferentiated and PMA-induced differentiated MO3.13 cells. β-actin served as a loading control. **(F)** Representative confocal images of PHGDH expression in PMA-induced differentiated MO3.13 cells after OGD/R treatment. Scale bar, 10 μm. The data are means ± S.D., for all panels: *P < 0.05, **P < 0.01, ***P < 0.001, n. s., no significance by Two-Way ANOVA analysis followed by Bonferroni Test **(B,C)** and Student’s t-test **(D)**. All data are representative of or combined from at least three independent experiments.

We then investigated the molecular mechanism by which PHGDH-mediated serine synthesis reduces oligodendrocyte death after ischemic injury. We first examined whether PHGDH inhibition directly affected oligodendrocytes survival after OGD/R treatment. We used PMA to stimulate MO3.13 cells, a human oligodendrocytic cell line that is widely used to study oligodendrocytes function, to induce their differentiation into mature oligodendrocytes. These differentiated cells were subjected to OGD treatment and then treated with either vehicle or NCT-503 to assess effects on cell viability (Figure 6A). Consistent with our *in vivo* findings, PHGDH inhibition significantly increased oligodendrocytes death after OGD/R treatment compared with vehicle-treated groups, whereas it did not affect cell viability under normal culture conditions ( Figure 6B). However, NCT-503 failed to alter cell viability of undifferentiated MO3.13 cells, which resemble immature oligodendrocytes (Figure 6C). Moreover, we used PHGDH-specific targeting siRNA to knock down PHGDH expression, and PHGDH knockdown also reduced cell viability in PMA-stimulated cells compared to siNC-transfected controls after OGD/R (Figure 6D). Therefore, mature oligodendrocytes might depend on PHGDH-mediated serine synthesis for cell survival, while immature oligodendrocytes did not require PHGDH-mediated serine synthesis for survival after OGD/R.

The differential requirement for serine in the survival of immature versus mature oligodendrocytes prompted us to hypothesize that immature oligodendrocytes might rely on extracellular serine obtained from other cell types, while mature oligodendrocytes depend on intracellular *de novo* serine synthesis. Consistent with our hypothesis, the serine transporter SLC1A4 was highly expressed in undifferentiated MO3.13 cells, but poorly expressed in differentiated MO3.13 cells (Figure 6E). In addition, PHGDH was predominantly localized in the cytoplasm of PMA-differentiated MO3.13 cells after OGD/R treatment indicating the metabolic regulation of PHGDH (Figure 6F). Therefore, PHGDH inhibition could specifically affect oligodendrocyte death after OGD/R treatment *in vitro*.

### 3.7 PHGDH-mediated serine synthesis protects oligodendrocytes from death by increasing GSH and NADPH production to resist oxidative stress

**FIGURE 7:**
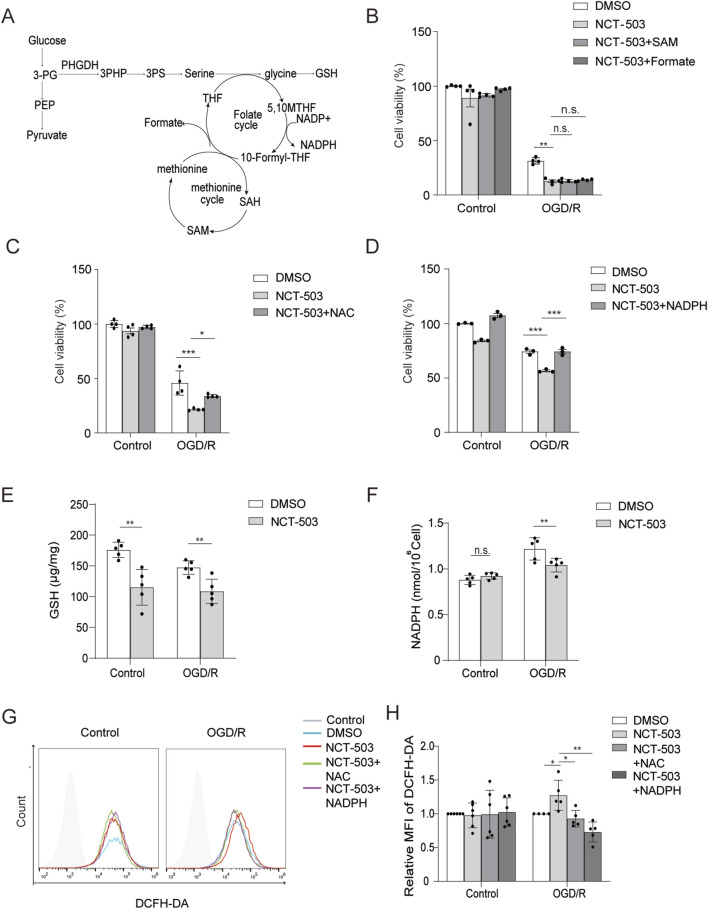
PHGDH-mediated serine synthesis facilitates oligodendrocyte survival through increased GSH and NADPH production to resist oxidative stress. **(A)** PHGDH-mediated serine synthesis and downstream metabolic influx. **(B)** Cell viability of PMA-induced MO3.13 cells measured by CCK8 assay of DMSO, NCT-503, NCT-503 + SAM and NCT-503 + Formate treatment. **(C,D)** Cell viability of MO3.13 cells measured by CCK8 assay of DMSO, NCT-503, NCT-503 + NAC treatment **(C)** or NCT + NADPH treatment **(D)**. n = 4 for each group. **(E,F)** Quantification of the abundance of intracellular GSH **(E)** and NADPH **(F)** in PMA-induced MO3.13 cells. n = 5 for each group. **(G,H)** Flow cytometry analysis **(G)** and quantification **(H)** of DCFH-DA in PMA-induced MO3.13 cells subjected to OGD/R treatment for 3.5 h and reperfusion for 12 h in the presence of DMSO, NCT-503, NCT503 plus NAC or NADPH. n = 6 (Control) and n = 5 (OGD/R) for each group. The data are means ± S.D., for all panels: *P < 0.05, **P < 0.01, ***P < 0.001, n. s., no significance by Two-Way ANOVA analysis followed by Bonferroni Test **(B–F,H)**. All data are representative of or combined from at least three independent experiments.

As PHGDH-mediated serine synthesis regulates downstream metabolic influx (Figure 7A), we wondered whether the well-established downstream metabolites of serine metabolism including SAM, glutathione (GSH), formate (a one-carbon metabolite derived from serine catabolism) and nicotinamide adenine dinucleotide phosphate (NADPH) affect oligodendrocyte death. Notably, addition of SAM and formate did not rescue the reduced survival of differentiated MO3.13 cells in the presence of NCT-503 after OGD/R insult (Figure 7B). In contrast, supplementation with N-acetylcysteine (NAC), a precursor of GSH, or exogenous NAPDH, the two well-known antioxidants, obviously restored the survival of differentiated MO3.13 cells after OGD/R treatment (Figures 7C,D). Consistent with these findings, PHGDH inhibition reduced the intracellular accumulation of GSH and NADPH (Figures 7E,F). These results suggest that PHGDH-mediated serine synthesis promotes oligodendrocyte survival by enhancing the production of GSH and NADPH.

We further tested whether PHGDH-mediated accumulation of GSH and NADPH protects oligodendrocytes from OGD/R-induced death by reducing oxidative stress. Reactive oxygen species (ROS) analysis showed that NCT-503 treatment significantly increased ROS levels, whereas treatment with NAC or NADPH reduced ROS levels to those observed in the control group (Figures 7G,H). Collectively, these data demonstrated that PHGDH promoted oligodendcocyte survival by reducing oxidative stress via NADPH and GSH accumulation.

## 4 Discussion

White matter is highly vulnerable to early ischemic injury, and oligodendrocyte survival is critical for maintaining myelin integrity and supporting long-term functional recovery (McIver et al., 2010; Wang et al., 2016). While cellular metabolism is profoundly altered after brain ischemia due to insufficient glucose and nutrient supply, how metabolism contributes to oligodendrocyte survival remains largely unknown. In this study, we found that PHGDH-mediated *de novo*serine synthesis directly promotes oligodendrocyte survival by enhancing GSH and NADPH production via one-carbon metabolism, thereby reducing oxidative stress and facilitating functional recovery after ischemic stroke (related to Figure 7).

Previous studies have demonstrated the importance of glycolysis and lipid metabolism in oligodendrogenesis (Narine and Colognato, 2022). The role of amino acid metabolism in oligodendrocyte survival after ischemia is largely unknown. Here, we demonstrate that PHGDH-driven L-serine synthesis in oligodendrocytes helps maintain the oligodendrocyte population after ischemic injury, likely by mitigating oxidative stress (related to Figure 7). Although L-serine is known to be neuroprotective in ways including promoting neuronal synaptic activity (Wang et al., 2010; Ye et al., 2021), enhancing cerebral blood flow (CBF) (Ren et al., 2013), facilitating OPCs proliferation and the survival and differentiation of neural stem cells (NSCs) (Metcalf et al., 2018; Sun et al., 2014) as well as inhibiting neuroinflammation (Wang et al., 2019; Zhai et al., 2015). Our data showed that PHGDH inhibition with NCT-503 directly and selectively affects mature oligodendrocytes without altering neuronal or OPC viability (related to Figures 2, 4, 6, 7). These data collectively demonstrate the complex neuroprotective roles of serine after brain injury. Further investigation is needed to clarify how PHGDH-mediated *de novo*serine synthesis contributes to different brain diseases and how other amino acid metabolic pathways influence injury and recovery.

In this study, we found that PHGDH is selectively expressed in oligodendrocytes and astrocytes and barely detectable in neurons, microglia and OPCs (related to Figure 1). This raises the question of how serine sources differ among cell types as serine can be imported from the extracellular space or synthesized via the PHGDH pathway (Shen et al., 2021; Yang and Vousden, 2016). Neurons cannot synthesize L-serine *de novo*; PHGDH deficiency in mice leads to brain malformations (Furuya, 2008; Yoshida et al., 2004) owing to defective neurogenesis indicating that endogenously synthesized L-serine is essential for embryonic development (Kawakami et al., 2009). PHGDH supplies L-serine to neurons, where it is converted to D-serine and modulates NMDA receptor-mediated synaptic activity (Neame et al., 2019). Exported L-serine from glia serves as a neurotransmitter precursor (Maugard et al., 2021; Wolosker, 2011; Wolosker et al., 1999), consistent with our observation that PHGDH is absent in neurons and that PHGDH inhibition does not affect neuronal viability after ischemia. In addition to astrocytes, oligodendrocytes also provide metabolic and functional support to neurons (Tiane et al., 2019). Oligodendrocytes also provide metabolic support to neurons, but whether oligodendrocyte-derived serine contributes to synaptic activity *in vivo* remains unknown.

In addition to neurons, NCT-503 treatment did not influence immature oligodendrocytes viability. The expression pattern of the serine transporter SLC1A4 supports this distinction: immature oligodendrocytes express high levels, whereas mature oligodendrocytes express low levels. OPCs in normal mouse brains show little PHGDH expression (related to Figure 1 and Figrue 6). Differentiation into mature, myelinating oligodendrocytes is a multistep process regulated by specific growth and transcription factors, accompanied by metabolic reprogramming to meet bioenergetic and biosynthetic demand (Hughes and Stockton, 2021; Tiane et al., 2019). Our data reveal distinct serine sources for OPCs, immature, and mature oligodendrocytes, likely reflecting their different stages of functional specialization.

Astrocytes contribute to brain development, brain function maintenance and pathological changes (Hasel and Liddelow, 2021). Some studies showed that PHGDH is highly expressed in astrocytes (Chen X. et al., 2022; Lv et al., 2025). Our previous study demonstate that PHGDH facilitates astrocytes-mediated neuroinflammation and regulates brain injury after MPTP injection (Lv et al., 2025). In this study, we also found the localization of PHGDH in astrocytes and inhibition of PHGDH reduced astrocytes activation (related to Supplementary Figure S3) although which may not lead to the comprised functional behavior after PHGDH inhibition.

Previous studies showed that PHGDH-mediated serine synthesis contributes to both tissue development (Stegen et al., 2022; Vandekeere et al., 2018; Wang et al., 2022) and disease progression such as cancer (Rossi et al., 2022; Zhao et al., 2021), metabolic diseases (Chen H. et al., 2022; Sim et al., 2020) and infections (Shan et al., 2022; Shen et al., 2021). In this study, we revealed the role of PHGDH-mediated *de novo* synthesis of serine in functional recovery after ischemic stroke (related to Figures 2, 5). Currently, the impact of PHGDH-mediated serine synthesis can be achieved by promoting the generation of downstream metabolites involved in one-carbon metabolism including nucleotides, glutathione, S-adenosylmethionine (SAM) and NADPH (Ganini et al., 2021; Yang and Vousden, 2016), although some studies also depicted the serine-independent role of PHGDH (Ma et al., 2021; Shu et al., 2022). In our study, supplementing SAM or formate failed to rescue oligodendrocyte death caused by PHGDH inhibition, whereas the antioxidants NAC and NADPH reversed the loss of cell viability, highlighting a serine-dependent, oxidative stress–mediated mechanism. These results demonstrate that PHGDH supports oligodendrocyte survival and functional recovery after stroke through a specific, metabolism-driven pathway.

### 4.1 Limitation of the study

In this study, we employed a pharmacological inhibitor of PHGDH to suppress its activity in the MCAO model. While this approach effectively reduces PHGDH function, genetic conditional depletion of PHGDH specifically in oligodendrocytes would provide a more precise and acute demonstration of its role in maintaining white matter integrity. Furthermore, employing primary oligodendrocyte cultures could allow direct assessment of PHGDH inhibition on oligodendrocyte survival following ischemic injury, thereby complementing *in vivo* findings and strengthening causal inference.

## Data Availability

The original contributions presented in the study are included in the article/Supplementary Material, further inquiries can be directed to the corresponding authors.
