# Supplementary material for: PHGDH-mediated serine synthesis reduces oligodendrocyte death by sustaining GSH and NADPH levels after brain ischemia
Source: Front Pharmacol. 2026 Apr 15;17:1735477. doi: 10.3389/fphar.2026.1735477 (PMC13124935; doi:10.3389/fphar.2026.1735477)
Supplement: Supplementary file 1 [file DataSheet1.pdf]

Supplementary Figure legends

Supplementary Figure 1. NCT-503 does not affect sensorimotor and cognitive function in physiological conditions.

(A) Schematic of experimental timeline. (B-D) Neurological function analysis after vehicle and NTC-503 treatment in normal conditions by adhesive removal test (B), rotarod tests (C) and NOR test (D). n=6 for each group. The data are means ± S.D. for all panels: n.s., no significance by Student's T-test.

Supplementary Figure 2. Effects of PHGDH inhibition on neuronal viability.

(A) Cell viability of DMSO or NCT-503 treated neurons after OGD/R treatment or in normal culture conditions. n=6 for each group. The data are means ± S.D. for all panels: ***P<0.001, n.s., no significance by Student's T-test (A).

Supplementary Figure 3. Effects of PHGDH inhibition on astrocytes.

(A) Cell viability of primary cultured astrocytes in the presence of DMSO or NCT-503. n=9 for each group. (B) Representative immunofluorescence images and statistical analysis of GFAP intensity in the cortex of mice brains injected with vehicle or NCT-503 after tMCAO for 3 days. Scale, 20 μm, n=7. The data are means ± S.D. for all panels: ***P<0.001, n.s., no significance by Student's T-test (A-B).

Figure S1

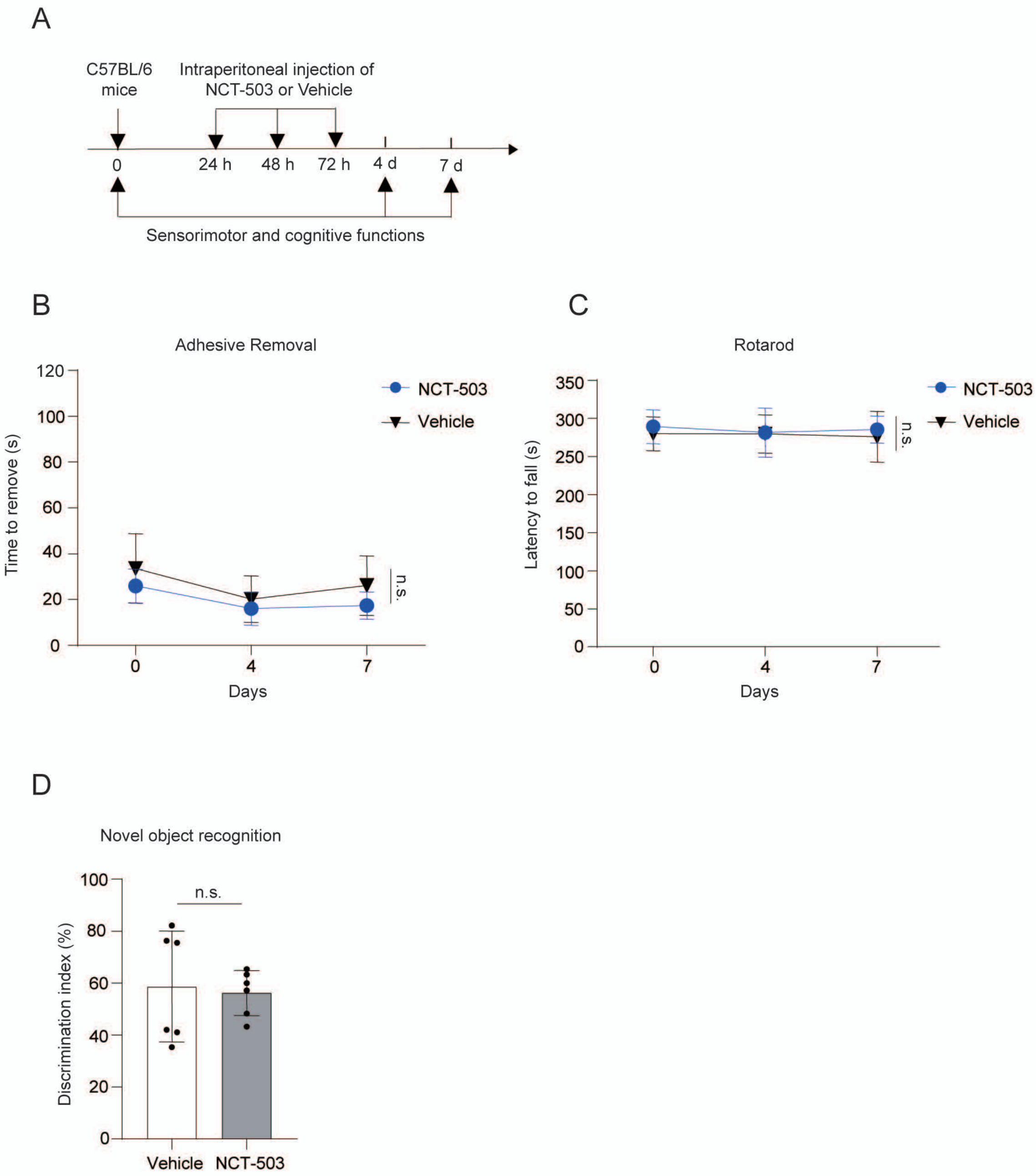
A
C57BL/6 mice
Intraperitoneal injection of NCT-503 or Vehicle
0
24 h
48 h
72 h
4 d
7 d
Sensorimotor and cognitive functions
B
Adhesive Removal
Time to remove (s)
120
100
80
60
40
20
0
0
4
7
Days
NCT-503
Vehicle
n.s.
C
Rotarod
Latency to fall (s)
350
300
250
200
150
100
50
0
0
4
7
Days
NCT-503
Vehicle
n.s.
D
Novel object recognition
Discrimination index (%)
100
80
60
40
20
0
n.s.
Vehicle
NCT-503

Figure S2

A

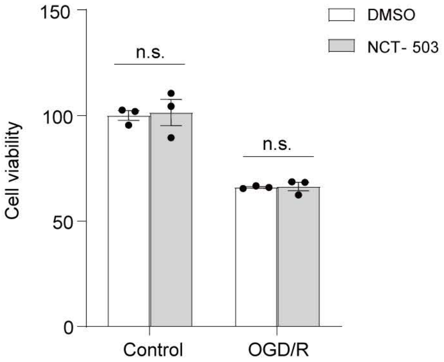

Figure S3

A

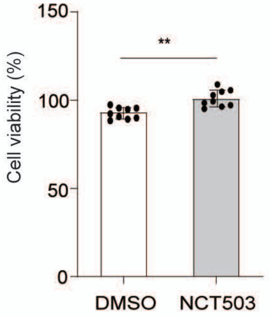

Cell viability (%)
150
100
50
0
**
DMSO
NCT503

B

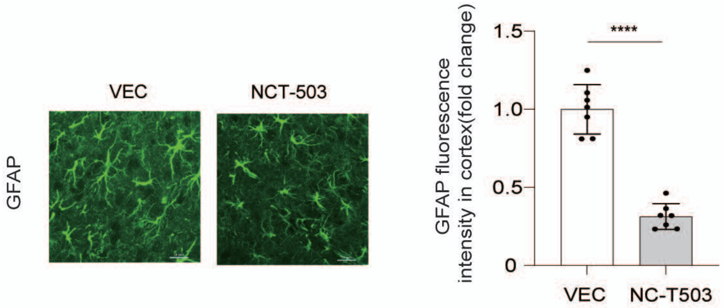

VEC
NCT-503
GFAP
GFAP fluorescence intensity in cortex(fold change)
1.5
1.0
0.5
0
****
VEC
NC-T503
